# Sex Hormone-Binding Globulin Is Associated with Obesity and Dyslipidemia in Prepubertal Children

**DOI:** 10.3390/children7120272

**Published:** 2020-12-04

**Authors:** Gihong Park, Kyungchul Song, Youngha Choi, Jun Suk Oh, Han Saem Choi, Junghwan Suh, Ahreum Kwon, Ho-Seong Kim, Hyun Wook Chae

**Affiliations:** Department of Pediatrics, Severance Children’s Hospital, Endocrine Research Institute, Yonsei University College of Medicine, Seoul 03722, Korea; onenewman@yuhs.ac (G.P.); endosong@yuhs.ac (K.S.); youngha@yuhs.ac (Y.C.); joojang87@yuhs.ac (J.S.O.); hansaem6890@yuhs.ac (H.S.C.); suh30507@yuhs.ac (J.S.); armea@yuhs.ac (A.K.); kimho@yuhs.ac (H.-S.K.)

**Keywords:** sex hormone-binding globulin, obesity, dyslipidemias, lipids, child

## Abstract

Sex hormone-binding globulin (SHBG) is associated with age, sex, and puberty. The association of SHBG with various diseases has been suggested nowadays, however, the relationships in prepubertal children have not been sufficiently investigated. This study analyzed the relationship of SHBG with body mass index (BMI) and plasma lipid levels in prepubertal children. We evaluated the association of SHBG with BMI among the 693 prepubertal children subdivided into normal, overweight, and obese groups, with plasma lipid levels among the children subdivided into normal and dyslipidemia groups. The obese and overweight group had lower SHBG levels than the normal BMI group in both sexes. The dyslipidemia group included subjects with low high-density lipoprotein cholesterol (HDL-C), high triglycerides (TG), or a high atherogenic index of plasma (AIP); this group had lower SHBG than the normal lipid group. SHBG was positively correlated with HDL-C, and negatively correlated with TG and AIP. After adjusting for BMI, SHBG was positively correlated with HDL-C and negatively correlated with TG and AIP in all groups. In conclusion, SHBG levels are closely correlated with BMI in prepubertal children. SHBG may play a meaningful role in the decrease in HDL-C and increase in TG during prepubertal age.

## 1. Introduction

Sex hormone-binding globulin (SHBG), a circulating glycoprotein that binds testosterone and estradiol with high affinity, is produced primarily in the hepatocytes of the liver [1]. Regulation of SHBG is associated with various factors including age, puberty, obesity, and dietary factors. SHBG levels rise after birth, remain stable during childhood, and decrease after pubertal onset [2]. In addition, SHBG levels are negatively associated with adiposity and chronic inflammation as well as a high-protein diet [3,4].

The nuclear receptor hepatic nuclear factor-4α (HNF4α) plays a key role in lipid metabolism. Functional HNF4α-binding sites are found in over 140 genes involved in the metabolism of glucose and lipids and in the proximal promoter of the SHBG gene [2]. Therefore, SHBG has also been associated with various metabolic disorders. A retrospective study of 141 men reported that lower SHBG levels were related to a higher prevalence of metabolic syndrome [5]. Another study reported that SHBG levels were negatively correlated with a risk of type 2 diabetes [6]. Severities of non-alcoholic fatty liver disease were also negatively related to SHBG levels in a study of adult patients with type 2 diabetes [7]. Low plasma SHBG level was suggested as a risk factor of cardiovascular disease in adult studies [8,9]. In addition, a study of 180 postmenopausal women showed that SHBG was positively correlated with high-density lipoprotein cholesterol (HDL-C) and negatively correlated with low-density lipoprotein cholesterol (LDL-C) [10].

In children, very few studies have investigated the association between SHBG and obesity and/or lipid profiles [11,12,13,14]. To the best of our knowledge, investigations of the association between SHBG and obesity and dyslipidemia are very limited in prepubertal children.

This study investigated the association of SHBG with body mass index (BMI) and plasma lipid levels in prepubertal children. The aim of this study was to test the following hypotheses: (1) SHBG may be affected by children’s BMI; and (2) SHBG may be associated with changes in lipid profiles.

## 2. Materials and Methods

### 2.1. Study Population

**Figure 1:**
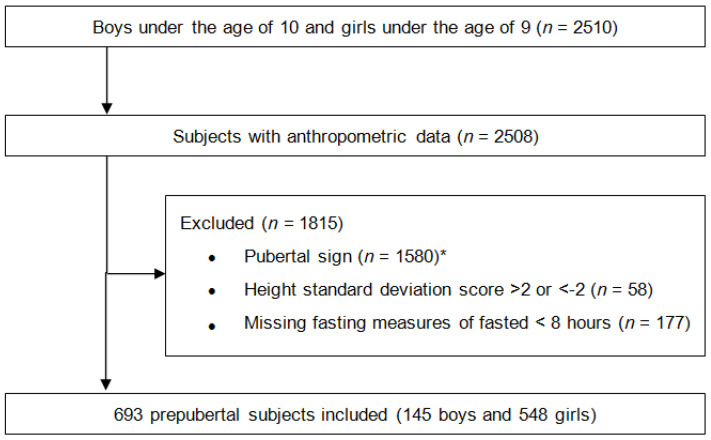
Flowchart of study population. * Objective breast development at ≥ Tanner stage 2 in girls and testicle development at ≥4 mL in boys, and/or luteinizing hormone concentrations of ≥0.2 IU/L, and/or bone age advancement compared to chronological age.

Figure 1 shows the study design and exclusion of subjects. This study was performed in accordance with the Declaration of Helsinki. The Institutional Review Board of Yonsei University Gangnam Severance Hospital approved this study and waived the requirement to obtain informed consent from the study participants (No. 3-2019-0203). Among 2510 children, 693 prepubertal children were included (145 boys and 548 girls). Pubertal signs included objective breast development at ≥Tanner stage two in girls and testicle development at ≥4 mL in boys, and/or luteinizing hormone concentrations of ≥0.2 IU/L, and/or bone age advancement compared to chronological age.

### 2.2. Data Collection

Height was measured by a stadiometer (Harpenden Ltd., Crymych, UK) to the nearest 0.1 cm. Weight was measured using a digital scale with a precision of 0.1 kg (SECA, model 707). BMI was calculated as weight (kilograms) divided by height (meters) squared. The height, weight, and BMI were presented as SDS on the basis of the 2017 Korean National Growth Charts [15]. Children were classified as normal weight (<85th percentile), overweight (85th to <95th percentile), or obesity (≥95th percentile) according to BMI percentile.

Following an 8-h fast, blood samples were collected, processed, and immediately refrigerated. SHBG level was determined by an immunofluorescence assay (Delfia, Wallac Oy, Turku, Finland). LDL-C level was calculated with the Friedewald formula [16], (LDL-C = total cholesterol (TC) − [HDL-C + (Triglycerides (TG)/5)]), and non-HDL-C was calculated by subtracting the HDL-C value from the TC value [17]. The cut-off of each dyslipidemia criterion was defined according to the American Academic of Pediatrics guideline, as follows: TC, ≥200 mg/dL; LDL-C, ≥130 mg/dL; TG, ≥100 mg/dL; HDL-C, <40 mg/dL; and non-HDL, ≥145 mg/dL [18]. An atherogenic index of plasma (AIP), defined as log(TG/HDL-C), of >0.15 was considered an abnormal value [19].

We evaluated the association of SHBG with BMI among the children subdivided into normal, overweight, and obese groups, and with plasma lipid levels among the children subdivided into normal and dyslipidemia groups. Moreover, we investigated the association between SHBG and plasma lipid levels after adjusting for BMI.

### 2.3. Statistical Analysis

Subgroup analyses with independent *t*-tests and analysis of variance were performed to compare the clinical parameters. Post hoc analyses were carried out with Bonferroni test. To determine the association between SHBG and plasma lipid levels, Pearson correlation, univariate linear regression, and multivariable regression analyses were used, and the correlation was displayed with a scatter plot and fitted line. Significance was determined as *p* < 0.05. The data were analyzed using SPSS version 25.0 (SPSS Inc., Chicago, IL, USA).

## 3. Results

### 3.1. Comparison of SHBG in the Children by BMI

**Figure 2:**
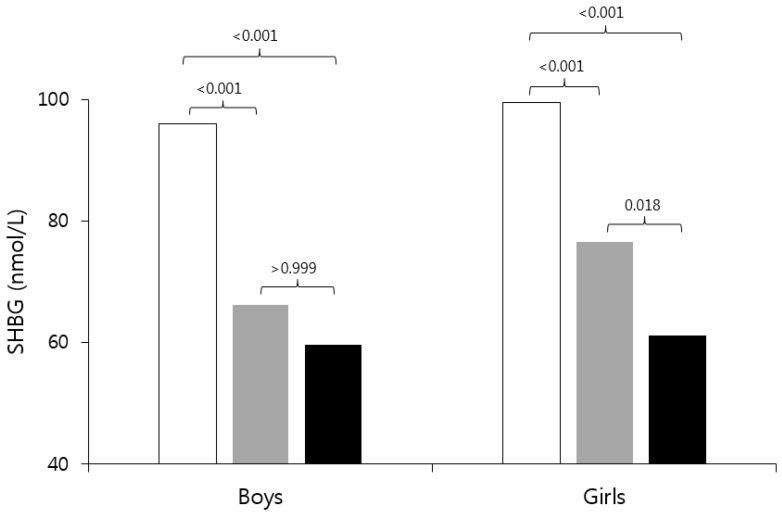
Comparison of sex hormone-binding globulin (SHBG) in the prepubertal children by BMI. The “white” bar is the normal group, the “gray” bar is the overweight group, and the “black” bar is the obese group. The number on the bar is the *p*-value of the Bonferroni test. SHBG: sex hormone-binding globulin; BMI: body mass index.

Baseline characteristics of participants according to sex and BMI are shown in Table 1. In boys, the obese group had higher TC, LDL-C, TG, non-HDL-C, and AIP than the normal BMI group. In girls, the obese group had higher TG, non-HDL-C, and AIP and lower HDL-C than the normal BMI group, and the overweight group had higher TC, LDL-C, TG, non-HDL-C, and AIP and lower HDL-C than the normal BMI group. The obese and overweight groups had lower SHBG levels than the normal BMI group in both sexes. In addition, the obese group had lower SHBG than the overweight group in girls (Figure 2).

### 3.2. Comparison of SHBG among Dyslipidemia and Normal Lipid Level Groups in Children

**Figure 3:**
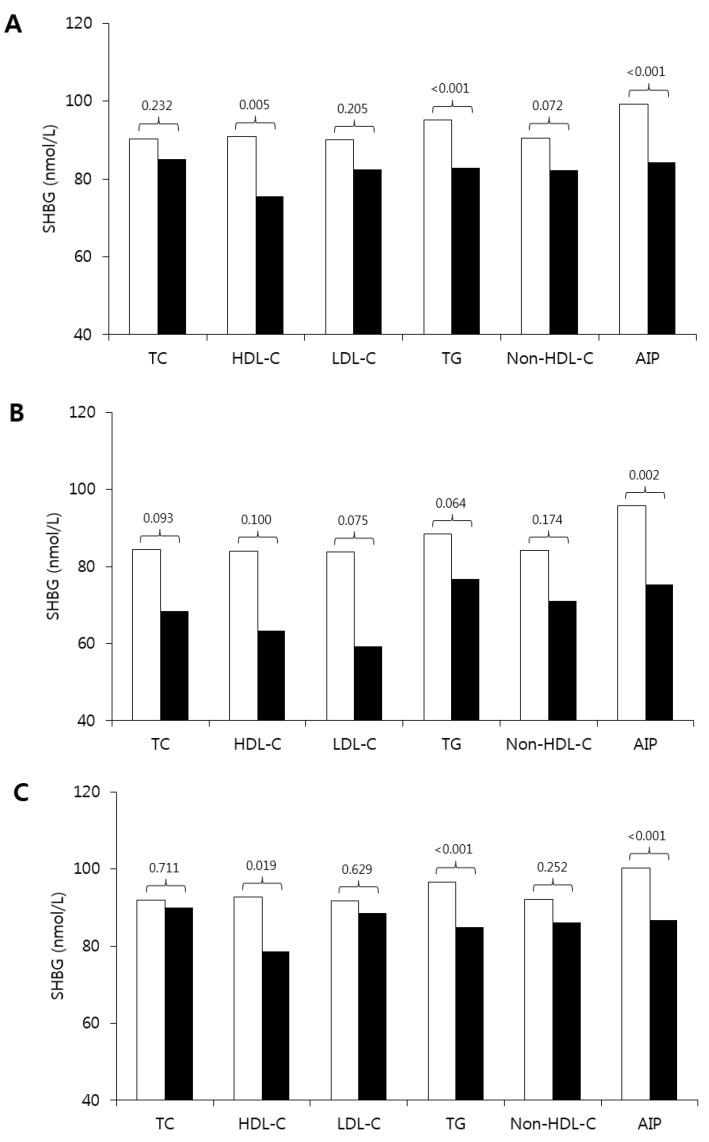
Comparison of SHBG among dyslipidemia and normal lipid profile groups in prepubertal children (**A**), boys (**B**), and girls (**C**). The “white” bar is the normal lipid group and the “black” bar is the dyslipidemia group. The number on the bar is the *p*-value. The criteria for dyslipidemia: TC, ≥200 mg/dL; LDL-C, ≥130 mg/dL; TG, ≥100 mg/dL; HDL-C, <40 mg/dL; non-HDL, ≥145 mg/dL; and AIP, >0.15. SHBG: sex hormone-binding globulin; TC: total cholesterol; HDL-C: high-density lipoprotein cholesterol; LDL-C: low-density lipoprotein cholesterol; TG: triglycerides; AIP: atherogenic index of plasma.

Figure 3 shows the comparison of SHBG levels between the normal and dyslipidemia groups. When all subjects were included in the analysis, SHBG was lower in the groups with lower HDL-C and higher TG and AIP than the normal group. The result of the mean difference between the normal group and the dyslipidemia group was different according to sex. In girls, SHBG was lower in the groups with low HDL-C, high TG, and high AIP. In boys, the results showed a similar pattern, however, a significant difference was shown in the AIP level.

### 3.3. Association of SHBG with Lipid Profiles

Table 2 shows the univariate and multivariable linear regression models for lipid profiles as dependent variables. Univariate linear regression analyses showed a positive correlation of SHBG with HDL-C and a negative correlation of SHBG with TG and AIP in all groups. Non-HDL-C was negatively correlated with SHBG in the total group and among boys. In multivariable linear regression analyses after adjusting for BMI SDS (standard deviation score), SHBG was positively correlated with HDL-C and negatively correlated with TG and AIP in all groups. The coefficients of determination were higher in boys than in girls. In addition, TC and LDL-C were positively correlated with SHBG in the total group and among girls.

**Figure 4:**
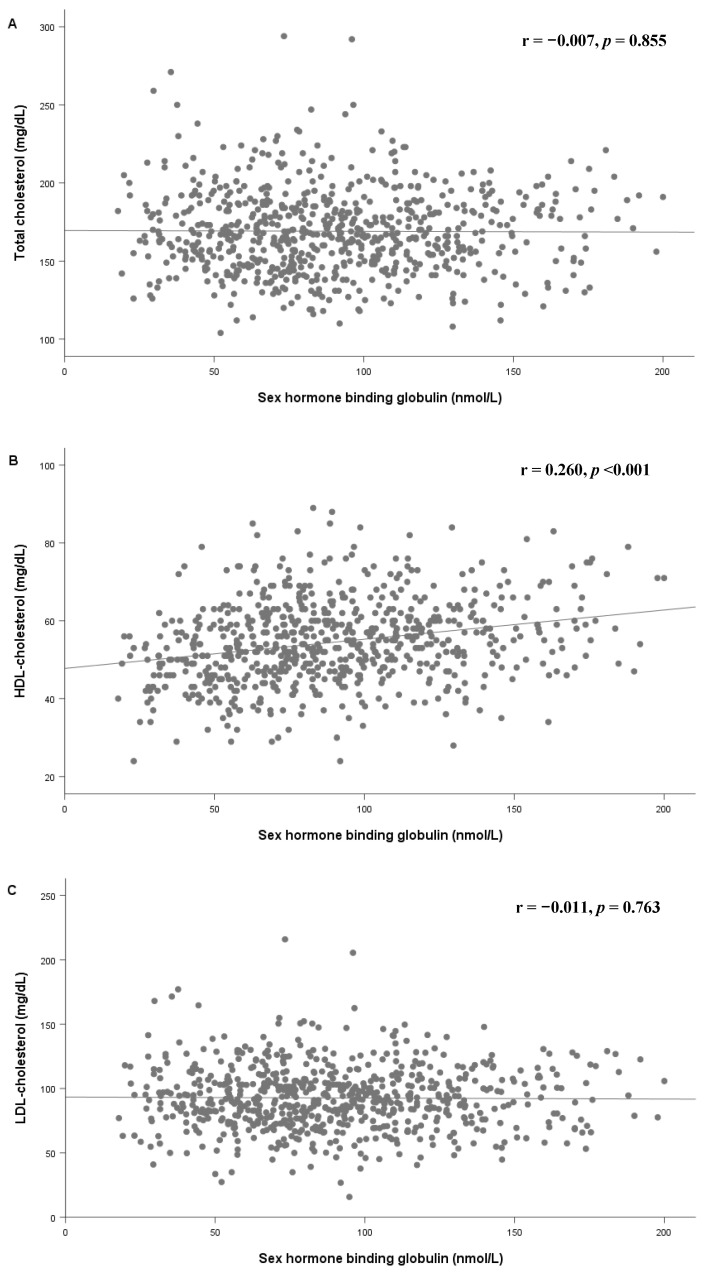

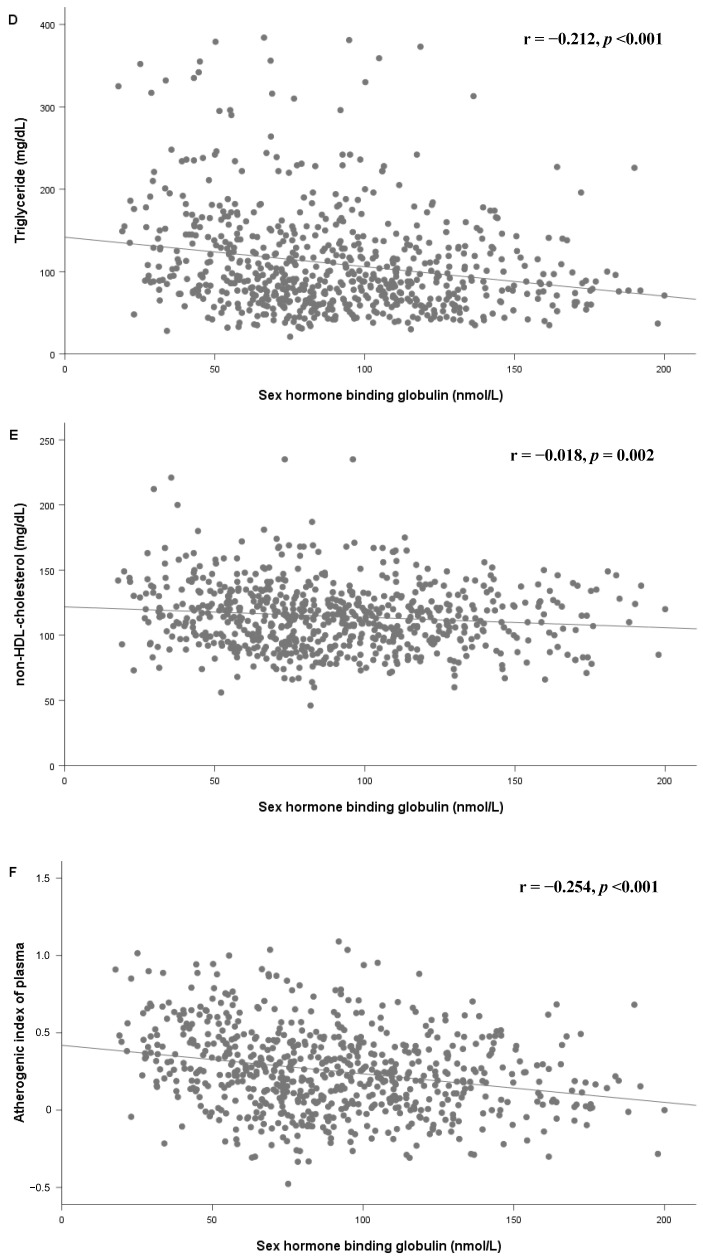
Scatter plot and fitted line of SHBG and total cholesterol (**A**), HDL-C (**B**), LDL-C (**C**), triglycerides (**D**), non-HDL-C (**E**), and Atherogenic index of plasma (**F**) in all subjects. SHBG: sex hormone-binding globulin; HDL-C: high-density lipoprotein cholesterol; LDL-C: low-density lipoprotein cholesterol.

Figure 4 shows the scatter plot and fitted line of SHBG and lipid profiles. HDL-C increased with an increase in SHBG, while TG, non-HDL, and AIP decreased with an increase in SHBG among prepubertal children.

## 4. Discussion

In this study, the higher BMI group showed significantly lower SHBG levels in both sexes. Our study showed a positive correlation between SHBG and HDL-C, and a negative correlation between SHBG and TG and AIP in prepubertal children. These relationships were present even after adjusting for BMI SDS, which was stronger in boys than in girls. The dyslipidemia group had lower SHBG levels than the normal group.

The lower SHBG level in the higher BMI group observed in this study is consistent with that of previous results of adult studies. A study of 141 men showed a negative correlation of SHBG with BMI [5]. A cross-sectional study of pubertral children reported that SHBG level was negatively correlated with BMI [20]. In addition, a prospective analysis of 1377 young adults reported that SHBG level was associated with future insulin resistance [21]. These studies suggested that insulin resistance in metabolic syndrome may decrease the SHBG level because insulin is a potent inhibitor of SHBG production in the liver [12]. Meanwhile, low SHBG levels in subjects with obesity may be associated with increased liver fat. Selva et al. reported that monosaccharide-induced lipogenesis inhibited hepatic SHBG expression in vitro and in mice [22]. In addition, Peter et al. found that liver fat was negatively associated with SHBG level, while total body or visceral fat was not significantly related to SHBG level [23]. SHBG levels were not different between overweight and obese children in our study. This lack of differences might be associated with the factors contributing to the SHBG level and/or obesity, such as pro-inflammatory markers and liver fat content [2,3].

The association of plasma lipid levels with sex hormones and SHBG has been suggested based on evidence of changes in sex hormones and plasma lipid profiles with sex and puberty. In a study of SHBG reference ranges, SHBG level increased gradually from the age of one to eleven years, and then decreased until 17 years of age in both sexes [24]. Another study found that SHBG decreased gradually from age six to twenty years in both sexes [25]. The reason for these changes is not clear but may be associated with the regulation of SHBG by androgen and estrogen [2].

Plasma lipid levels also change with pubertal development during the peripubertal period [12,26,27,28]. Bertrais et al. reported a negative correlation between advancement of Tanner stage and TC and a positive correlation between advancement of Tanner stage and TG [26]. In a study of pubertal children, older boys showed lower TC, HDL-C, LDL-C, and apolipoprotein A-I levels than younger boys [12]. Pinhas-Hamiel et al. reported that the advancement of Tanner stages was negatively correlated with TC, HDL-C, LDL-C, and non-HDL-C in children with obesity [27]. In a study of Korean children, children aged 12–18 years had lower TC than children aged ten to eleven years in both sexes, and pubertal boys had lower LDL-C and HDL-C than prepubertal boys [28].

The association between plasma lipid levels with sex hormones and SHBG has been investigated. In an adult study, testosterone and SHBG levels were negatively correlated with TG and positively correlated with HDL-C in men [29]. In a study of postmenopausal women, SHBG levels were positively correlated with HDL-C and negatively correlated with LDL-C and TG [10]. The association of SHBG with lipid profiles in children was investigated in very few studies. A population-based study with 370 Spanish pubertal children (ages 12 to 15) reported that SHBG was positively correlated with HDL-C and negatively correlated with log TG and apolipoprotein A-I [12]. A study of 84 children with obesity in Taiwan showed a positive correlation between SHBG and HDL-C [13]. These previous studies focused on pubertal children or were limited to children with a small sample size.

In adults and pubertal children, SHBG is thought to affect lipid levels through regulating sex hormones. HNF4α, which plays an important role in lipid metabolism, binding sites are found in the proximal promoter of the SHBG gene [2]. Among lipid genes, 43 genes are transcriptionally regulated by estrogen receptor alpha [30]. Thus, the loss of hepatocyte estrogen receptor alpha increases the expression of lipid synthesis genes [31]. In addition, androgen receptor knockout mice showed elevated plasma cholesterol and TG levels [32]. In prepubertal children, we might explain the association between SHBG and plasma lipid levels with the following hypotheses: (1) changes in sex hormones and SHBG levels prior to pubertal onset may affect changes in plasma lipid levels; and (2) insulin resistance in metabolic syndrome accompanied by dyslipidemia may alter SHBG levels. Further studies are necessary to explain the mechanism and causal relationship of SHBG with BMI and lipid profiles.

Our study has some limitations. First, it was a cross-sectional, single-center study in nature, thus a causal relationship of changes in SHBG with BMI and lipid profiles could not be established. Second, the number of male subjects was not sufficient and relatively small when compared to females. These factors limited the generalizability of the study results. Third, we could not include influencing factors such as lifestyle habits, diet differences, family history data of cardiovascular risk, and metabolic alteration. Further, we could not consider important markers in the pathogenesis of cardio-metabolic complications such as insulin levels, pro-inflammatory markers, hepatic lipid content, and oxidative stress [18,33,34,35]. Obesity increases oxidative stress, and it contributes to the pathogenesis of cardiovascular and metabolic complications [35]. In addition, SHBG level is negatively associated with hepatic lipid content and insulin resistance as well as pro-inflammatory markers [2,3]. Further large longitudinal studies including these factors are needed to reveal causal relationship.

Nevertheless, we investigated the association between SHBG level and plasma lipid levels in prepubertal children excluding the confounding effect of puberty. We included both obesity and normal BMI groups with a relatively large dataset.

## 5. Conclusions

SHBG levels are closely correlated with BMI in prepubertal children. This study showed that SHBG level was positively correlated with HDL-C levels and negatively correlated with TG and AIP as well as BMI. SHBG might play an important role in the regulation of HDL-C and TG and might be a meaningful marker for future cardiovascular risk in children.

## Figures and Tables

**Table 1:** Baseline characteristics of prepubertal children by sex and BMI.

	Total (*n* = 693)	Boys (*n* = 145)				Girls (*n* = 548)			
		Normal (*n* = 86)	Overweight (*n* = 27)	Obesity (*n* = 32)	*p* Value	Normal (*n* = 403)	Overweight (*n* = 82)	Obesity (*n* = 63)	*p* Value
Age (year)	8.03 ± 0.94	8.81 ± 0.91	8.71 ± 1.06	8.49 ± 0.99	0.277	7.93 ± 0.80	7.75 ± 0.88	7.46 ± 0.98	<0.001
Height (cm)	129.53 ± 7.19	135.07 ± 6.47	136.97 ± 6.38	135.00 ± 6.12	0.374	128.10 ± 6.38	128.11 ± 6.76	127.04 ± 7.40	0.484
Height SDS	0.29 ± 0.84	0.50 ± 0.86	0.96 ± 0.60	0.83 ± 0.68	0.013	0.12 ± 0.80	0.33 ± 0.86	0.46 ± 0.90	0.003
Weight (kg)	30.11 ± 6.32	32.35 ± 4.80	39.42 ± 5.23	43.29 ± 6.21	<0.001	26.87 ± 3.89	32.07 ± 4.15	34.52 ± 5.95	<0.001
Weight SDS	0.50 ± 0.95	0.25 ± 0.71	1.42 ± 0.27	2.03 ± 0.43	<0.001	0.03 ± 0.71	1.21 ± 0.40	1.78 ± 0.49	<0.001
BMI (kg/m^2^)	17.81 ± 2.58	17.65 ± 1.67	20.89 ± 1.15	23.63 ± 2.12	<0.001	16.31 ± 1.42	19.44 ± 0.77	21.21 ± 1.56	<0.001
BMI SDS	0.39 ± 1.11	0.01 ± 0.76	1.38 ± 0.15	2.34 ± 0.50	<0.001	−0.20 ± 0.77	1.33 ± 0.18	2.07 ± 0.36	<0.001
TC (mg/dL)	169.10 ± 26.61	161.14 ± 23.24	173.81 ± 34.75	184.19 ± 30.98	<0.001	167.21 ± 25.58	175.38 ± 29.39	174.21 ± 22.16	0.009
HDL-C (mg/dL)	54.50 ± 10.61	56.87 ± 12.53	53.04 ± 6.54	53.22 ± 13.26	0.179	55.34 ± 10.20	51.78 ± 9.81	50.75 ± 9.86	<0.001
LDL-C (mg/dL)	92.67 ± 24.72	82.53 ± 21.32	94.99 ± 32.33	101.47 ± 28.17	0.001	91.63 ± 23.28	99.28 ± 27.70	99.11 ± 23.58	0.005
Triglycerides (mg/dL)	109.65 ± 62.21	108.67 ± 72.43	128.78 ± 71.72	147.31 ± 82.04	0.039	101.27 ± 54.61	121.59 ± 69.90	121.67 ± 55.48	0.001
Non-HDL-C (mg/dL)	114.59 ± 24.93	104.27 ± 19.27	120.75 ± 35.45	130.93 ± 29.72	<0.001	111.86 ± 23.59	123.60 ± 26.41	123.44 ± 20.04	<0.001
AIP	0.25 ± 0.27	0.23 ± 0.28	0.32 ± 0.26	0.39 ± 0.30	0.015	0.22 ± 0.26	0.32 ± 0.26	0.35 ± 0.25	<0.001
Glucose (mg/dL)	92.56 ± 7.13	94.10 ± 6.08	94.15 ± 5.85	95.88 ± 5.20	0.327	91.80 ± 7.48	92.35 ± 7.06	93.24 ± 6.84	0.326
BUN (mg/dL)	12.33 ± 2.65	12.49 ± 2.63	13.36 ± 2.02	12.98 ± 2.24	0.232	12.04 ± 2.63	12.50 ± 2.58	13.02 ± 3.01	0.016
Creatinine (mg/dL)	0.44 ± 0.08	0.48 ± 0.08	0.47 ± 0.09	0.48 ± 0.07	0.821	0.43 ± 0.08	0.45 ± 0.09	0.42 ± 0.08	0.219
AST (IU/L)	27.41 ± 6.02	27.26 ± 5.45	27.19 ± 8.67	28.44 ± 8.82	0.691	27.73 ± 5.98	26.74 ± 4.62	26.02 ± 5.44	0.049
ALT (IU/L)	15.32 ± 10.08	15.66 ± 7.37	28.41 ± 31.47	25.34 ± 18.14	0.001	13.25 ± 4.57	14.66 ± 5.67	18.30 ± 10.03	<0.001

Values are presented as mean ± standard deviation. The *p*-value comes from the analysis of variance according to BMI among boys and girls, respectively. SHBG: sex hormone-binding globulin; BMI: body mass index; SDS: standard deviation score; TC: total cholesterol; HDL-C: high-density lipoprotein cholesterol; LDL-C: low-density lipoprotein cholesterol; AIP: atherogenic index of plasma; BUN: blood urea nitrogen; AST: aspartate transaminase; ALT: alanine transaminase.

**Table 2:** Univariate and multivariable linear regression models for lipid profiles as dependent variables in prepubertal children.

	TC (mg/dL)			HDL-C (mg/dL)			LDL-C (mg/dL)			Triglycerides (mg/dL)			Non-HDL-C (mg/dL)			AIP		
	Beta	R^2^	*p* Value	Beta	R^2^	*p* Value	Beta	R^2^	*p* Value	Beta	R^2^	*p* Value	Beta	R^2^	*p* Value	Beta	R^2^	*p* Value
Univariate linear regression with SHBG and BMI SDS on lipid profiles in prepubertal children																		
Total (*n* = 693)																		
SHBG	−0.005	<0.001	0.855	0.075	0.068	<0.001	−0.008	<0.001	0.763	−0.358	0.045	<0.001	−0.080	0.014	0.002	−0.002	0.064	<0.001
BMI SDS	4.254	0.032	<0.001	−1.747	0.034	<0.001	3.819	0.030	<0.001	10.816	0.037	<0.001	5.995	0.072	<0.001	0.056	0.054	<0.001
Boys (*n* = 145)																		
SHBG	−0.061	0.006	0.335	0.118	0.141	<0.001	−0.077	0.012	0.186	−0.511	0.065	0.002	−0.179	0.060	0.003	−0.002	0.103	<0.001
BMI SDS	6.295	0.065	0.002	−2.076	0.041	0.014	5.586	0.061	0.003	13.845	0.046	0.010	8.355	0.124	<0.001	0.061	0.060	0.003
Girls (*n* = 548)																		
SHBG	0.010	<0.001	0.740	0.065	0.053	<0.001	0.005	<0.001	0.851	−0.298	0.035	<0.001	−0.055	0.007	0.053	−0.002	0.052	<0.001
BMI SDS	3.863	0.026	<0.001	−1.816	0.036	<0.001	3.845	0.029	<0.001	9.081	0.029	<0.001	5.677	0.064	<0.001	0.053	0.049	<0.001
Multivariable linear regression models for lipid profiles as dependent variables after adjusting for BMI SDS																		
Total (*n* = 693)																		
SHBG	0.075	0.040	0.014	0.051	0.063	<0.001	0.064	0.036	0.027	−0.260	0.055	<0.001	0.011	0.072	0.692	−0.001	0.080	<0.001
Boys (*n* = 145)																		
SHBG	0.076	0.071	0.315	0.122	0.142	<0.001	0.034	0.063	0.621	−0.398	0.072	0.047	−0.045	0.127	0.520	−0.002	0.108	0.006
Girls (*n* = 548)																		
SHBG	0.078	0.035	0.022	0.065	0.072	<0.001	0.071	0.038	0.024	−0.221	0.044	0.003	0.026	0.065	0.398	−0.001	0.070	<0.001

SHBG: sex hormone-binding globulin; BMI: body mass index; SDS: standard deviation score; TC: total cholesterol; HDL-C: high-density lipoprotein cholesterol; LDL-C: low-density lipoprotein cholesterol; AIP: atherogenic index of plasma.
